# Root Canal Anatomy of Myanmar Permanent Mandibular Incisors in Mandalay Region

**DOI:** 10.1155/2020/8842636

**Published:** 2020-08-07

**Authors:** Nyan M. Aung

**Affiliations:** Department of Oral Biological Science, University of Dental Medicine (Mandalay), U Hla Htun's Hospice Cancer Foundation, Mandalay, Myanmar

## Abstract

**Introduction:**

Incomplete understanding of the root canal system leads to endodontic failure. Missed canal was the fourth most common endodontic failure, which needed retreatment. There were a few studies on internal morphology of posterior teeth of Myanmar population. However, there was no report on root canal anatomy of anterior teeth. So, the aim of the in vitro study was to investigate root canal anatomy of Myanmar permanent mandibular incisors in Mandalay Region by the staining and clearing method.

**Materials and Methods:**

A total of 96 teeth from the tertiary hospitals and one academic department in Mandalay Region were selected according to inclusion and exclusion criteria. Then, they were cleaned, drilled, stained with Indian ink, and decalcified with 5% nitric acid for 3 days. After that, they were dehydrated with ascending concentration of ethanol (80% overnight, 90% for 1 hour, and full strength for three hours). Finally, they were clarified with 98% methyl salicylate and investigated by calibrated observer in case of Vertucci's classification, allocation of apical foramen, and the detailed anatomy.

**Result:**

Almost 70% of the teeth had type Ι followed by 21.8% type ΙΙΙ, 4% 1-2-1-2-1 and type ΙΙ, and 1-3-2 and 2-3-1 comprised 1% each. Nearly 70% of apical foramen at central allocation of the root and 14% of detailed anatomy were seen in the sample teeth.

**Conclusion:**

Most of the sampled Myanmar teeth comprised one canal and one foramen followed by type ΙΙΙ. However, unusual anatomies, such as 1-2-1-2-1, 1-3-2, and 2-3-1, were also seen. Contrastively, proportions of central location of apical foramen and of detailed anatomy were differed from the former reports. This study summarized that 3 in 10 mandibular incisors comprised the evidence of second or third canal configuration. Three types of intercanal calcifications, fusiform, islet, and bead, were additionally explored.

## 1. Introduction

The Latin word ra'dicis den'tis is similar to the root canal. Around 200 BC, the oldest evidence was recognized as root canal therapy, which was found in the skull of Nabatean warriors in Israel [1]. However, undergoing treatment with incomplete understanding of this delicate anatomy looks like the vehicle running on the icy road. One example is the evidence of the second canal in permanent mandibular incisors, which leads to failure to notice. In addition, the missed canal was the 4^th^ most common endodontic failure, which needed retreatment [2].

Next to the most common type Ι (one canal and one foramen), permanent mandibular incisors comprised Vertucci's type ΙΙΙ configuration dominantly found in Chinese population [3], type ΙΙ in Italian population [4], and type V in some Turkish populations [5], all of which were CBCT studies. On the other hand, by the staining and clearing method, type ΙΙΙ was the second mode in different populations across the globe [6, 7]. And, opening of apical foramen is the clinical importance to be considered. Nearly eighty percent of this type of teeth had the apical foramen fenestrated at the side of the teeth [8]. Then, intricate anatomy, such as apical ramification, was commonly found in less than ten percent of the teeth in most situations [8].

First, internal anatomy of permanent molars of Myanmar population was investigated by Gulabivala and coworkers having used staining and clearing methods for more than a decade ago [9]. Also, Moe and colleagues found the anatomy of the mesial root of Burmese (Myanmar) mandibular first molar by means of micro-CT [10]. Among numerous methods to identify root canal anatomy of permanent dentition, canal staining and clearing are used to investigate three-dimensional canal configuration, the ability to detect detailed anatomy, its reliability (7), and economic concern. And, other methods, such as micro-CT, are not available in Myanmar and very expensive. Also, CBCT has low or no sensitivity to detect some categories of intricate anatomies [11].

In spite of the lack of evidence of root canal anatomy of Myanmar anterior teeth, there were only countable reports [9, 10] about the anatomy of Myanmar population. The internal morphology of the tooth group is needed to identify for both clinical and anthropological works to compare with the populations across the world, which can be global and national concerns. And, the missed canal was the fourth most common of endodontic failure, which needed retreatment mentioned previously [2]. The negligence of the root canal can be unexpectedly encountered in treating mandibular incisors. The most common endodontic failure among maxillary and mandibular anterior teeth in a particular population was detected in mandibular incisors [12].

More than 90% of the research expenditure was spent for only 10% of the world population [13]. This may benefit for people of high-income countries and can lead to health inequalities between rich and poor. So, the findings of this study can benefit for the people of low-middle income country.

So, the purpose of this in vitro study was to investigate root canal anatomy of Myanmar permanent mandibular incisors in Mandalay Region by the staining and clearing method.

Null hypothesis: there was no second canal anatomy in Myanmar permanent mandibular incisors in Mandalay Region.

## 2. Materials and Methods

This study was approved by the Research and Ethic Committee, University of Dental Medicine, Mandalay, Department of Human Resource for Health, Ministry of Health and Sports. REC number was M-17-2019, and approval number was Ethical/UDMM/M-17-2019.

However, the time frame allowed for the study was one year.

### 2.1. Sample Size Calculation

There was no previous report on root canal anatomy of Myanmar permanent mandibular incisors. So, the proportion of Vertucci's type Ι in previous study on another population was reliable to calculate sample size [6]. To fulfill the objectives, sample size was calculated by using the formula,

(1) $$\begin{array}{c} \;n=\frac{Z^{2}pq}{d^{2}}, \end{array}$$

*n* is the total number of permanent mandibular incisors required, *p* = 0.72 is the approximate value of the proportion or percentage of permanent mandibular incisors with one canal (Vertucci's type Ι) to be determined. *q* = 1−*p.*, and *Z*  is the standard normal distribution, if significance level = 5%, *z* = 1.96. *d* = precision = 0.09 (absolute precision).

(2) $$\begin{array}{c} n=\;1.96^{2}\times 0.72\times \frac{0.28}{0.09^{2}}=96. \end{array}$$

This study was an in vitro cross-sectional descriptive design. It was conducted in Department of Oral Biological Science, University of Dental Medicine (Mandalay) from 1^st^ September 2019 to 30^th^ December 2019.

### 2.2. Data Collection

From the three tertiary hospitals (Mandalay General Hospital, Oral and Maxillofacial Department (University of Dental Medicine, Mandalay), and 1000 bedded Nay Pyi Taw General Hospital, Nay Pyi Taw) and Department of Oral Biological Science (University of Dental Medicine, Mandalay) in Mandalay Region, the teeth were collected. The simple random sampling method was used.

### 2.3. Inclusion and Exclusion Criteria

Age, gender, ethnicity of the patients, position of tooth in the dental arch (left or right), and reasons for extraction were not considered. These potential confounding variables were out of the causal pathway and were excluded in this in vitro design study. Extracted permanent mandibular central and lateral incisors with recognizable crown morphology, complete root formation, and no previous dental treatment were included in this study. Congenital anomalies, root fracture, internal and external resorption, endodontically treated teeth, canal obstruction, and calcification were excluded by the naked eye and patency file (size 10#), and some were detected and excluded after staining and clearing procedure.

### 2.4. Study Procedure

96 extracted teeth were collected. First of all, the teeth were placed in 10% formalin before use for diaphanization and the small hole prepared on the lingual fossa of the teeth. Thereafter, teeth were immersed in 5% sodium hypochlorite solution for 24 hours to remove organic debris. Prior to decalcification, the teeth were washed in water for two hours. And then, China ink was injected through a small hole into the root canal. Meanwhile, negative pressure was applied at apical region of the teeth.

#### 2.4.1. Decalcification

And then, teeth were decalcified in 5% nitric acid (UDM Mandalay, Chemistry Department) for three days, and the acid was changed each day. The solution was agitated every 8 hours. After decalcification, the body of the root was bent by fingers (physical method) to check complete decalcification [14].

#### 2.4.2. Dehydration

After decalcification, the teeth were dehydrated in ascending concentration of ethanol (80% ethanol overnight, then 90% for 1 hour, and finally, 100% full strength for 3 hours). Finally, the apical third of tooth was transparent (the end point of dehydration) because the clarifying agent, methyl salicylate, can approach into the root only if the tooth was completely dehydrated.

#### 2.4.3. Clarification

After dehydration, the teeth were immersed in 98% methyl salicylate (AnalaR, BDH chemical limited Poole, UK) for 2 hours to make these transparent (the end point of clarification) because refractive index of methyl salicylate (wintergreen oil) is the same as that of the dentine. After clarification, three dimension of root canal anatomy can be identified in the transparent or cleared tooth.

Observer was calibrated according to Vertucci's classification. Cohen's kappa statistic for dichotomous variables was used for calibration (intraexaminer reproducibility). First, the expert who had 10 years experience in general dental practice and also 8 years of teaching experience in external and internal morphologies at University of Dental Medicine (Mandalay) observed and classified 10% of the specimen for calibration. And then, the observer was tested for these specimens (test 1). 2 weeks later, he was retested as previously mentioned (test 2). As a result, intraobserver agreement was obtained.

Finally, the anatomical type of root canal was identified according to Vertucci's classification. Fine details of the anatomy, such as location of apical foramen, presence or absence of lateral canal and accessory canal, and apical delta, were identified with domestically used ×3 magnifying glass.

The observer investigated the following anatomical features of the root canal system;

1. Root canal anatomy of the mandibular incisors according to Vertucci's classification
2. The location of apical foramen in relation to anatomic apex of the teeth, centrally or laterally
3. The occurrence of lateral canals, accessory canals, and apical delta in the teeth

### 2.5. Data Analysis

Initially, kappa score was calculated for intraobserver reproducibility (test-retest) for the observer in detecting Vertucci's classification. And, most of the parameters were presented with descriptive statistics such as frequency distribution tables along with percentages in the matter of number and types of root canal (Vertucci's classification/categorical variable), which are listed in Table 1, location of apical foramen, presence or absence of lateral canal, accessory canal, and apical delta (categorical variables). The number of root canal (categorical variable) was summarized according to main outcome of the study (Vertucci's classification). The data were analyzed by SPSS software.

## 3. Results

Intraobserver reproducibility or test-retest reliability (kappa statistic) was 0.737. This means that 73.7% agreement is between the two tested results of the principal observer.

**Figure 1:**
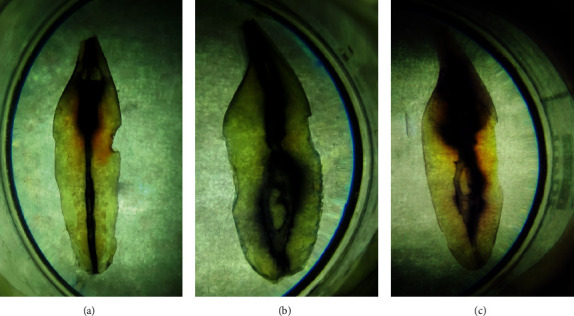
Vertucci's type Ι, type ΙΙΙ, and 1-2-1-2-1.

First, baseline distributions of types of mandibular incisors are provided in Table 2. Root canal anatomy of mandibular incisors according to Vertucci's classification is presented in Table 3. Locations of apical foramen in mandibular central and lateral incisors are listed in Table 4. Distribution of lateral canal, accessory canal, and apical delta in mandibular central and lateral incisors is described in Table 5. Distribution of number (%) of root canal in permanent mandibular central and lateral incisors is summarized in Table 6. Three common types (type Ι, ΙΙΙ, and 1-2-1-2-1) of root canal anatomy are shown in Figure 1.

**Figure 2:**
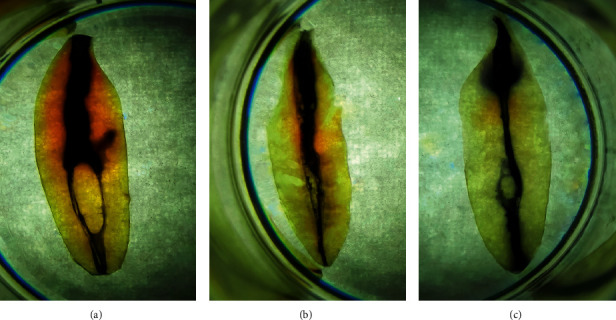
Three types of intercanal calcification: fusiform, islet, and bead.

**Figure 3:**
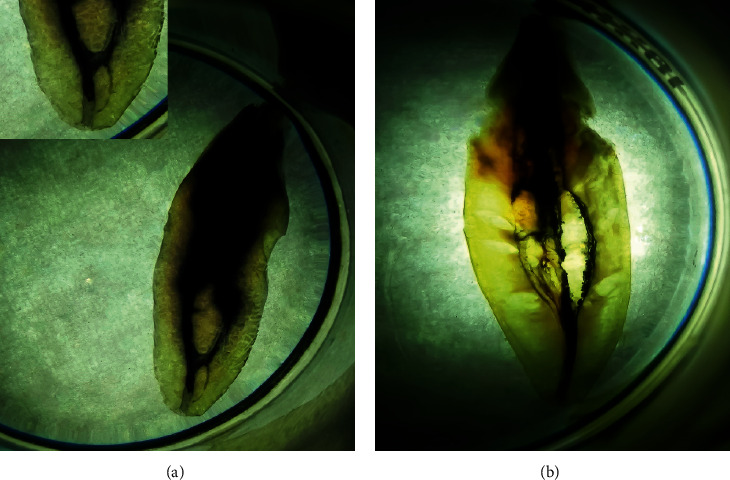
Loop in the position of accessory canal and 2-3-1 canal configuration.

As an exploratory analysis, there were three various form of intercanal calcification: fusiform, islet, and bead (Figure 2). Loop and 2-3-1 canal configuration are shown in Figure 3.

## 4. Discussion

The observer was calibrated to reduce observer bias, consequently misclassification bias, publication bias, and reader bias. 73.7% of intraobserver agreement was obtained in this study. Orwin stated that good agreement was in the range of 60%–74% [15].

More than 70% of the Myanmar permanent mandibular incisors in Mandalay Region comprised one canal and one foramen pattern, Vertucci's type Ι. However, nearly 3 out of 10 teeth from the sample were detected as two canal anatomy, which was the amalgamation of Vertucci's type ΙΙΙ, 1-2-1-2-1 (additional class), and Vertucci's type ΙΙ canal configurations. Altogether, 2.08% of the teeth was classified as the 1-3-2 and 2-3-1 aberrant canal system unpredictably. Moreover, 69.79% of the apical foramen opened at the center of root apex, whereas the rest of the specimens exposed lateral allocation (30.21%). Concordance to the final objective of the study, lateral canals, accessory canals, and apical delta were observed in nearly two out of ten teeth of the sample.

The most prevalent canal configuration in both maxillary and mandibular arches was stated as one canal with one foramen. In that case, the proportion of this prevalence in permanent mandibular incisors was varied from 55% [16] to 87.6% [17] in relation with the different populations. As a result, some of the evidences [6, 7] were complementary with the findings of this study.

The findings for clinical significance are the incidences of the second canal in permanent mandibular incisors differing among various ethnicities across the world. Of these, Vertucci's type ΙΙΙ canal configuration (1-2-1) was the predominantly found feature in permanent mandibular incisors in the majority of the world population [7, 16–18]. Our finding is consistent with these facts.

The most striking characteristic of this study is the third frequent type of the root canal system in Myanmar mandibular incisors. 1-2-1-2-1 canal configuration was detected as the third frequent mode participating in more than 4% of the study specimens. The outcome is contradictory to the former discoveries [7, 18]. In most of the ethnicities, type ΙΙΙ was followed by type ΙΙ or type V frequently. It seems to be an inadequate sample size to detect available evidence or also to be the anatomical significance of Myanmar population.

Aberrantly, both 2-3-1 and 1-3-2 were found in this study. Most of the studies with staining and clearing methods have no report associated with 3 canals in permanent mandibular incisors. However, some of the CBCT studies have reported these features. 1-3-1 canal configuration in the permanent mandibular lateral incisor was investigated by CBCT in Portugal population [19]. It appeared as the outliers of the data. After all, the finding from the in vitro method should not be comparable with that of in vivo.

Nearly 70% of apical foramina of the study specimens perforated at the center of the anatomic apex. This finding is inconsistent with data from previous study [16, 17]. At the same time, it has an agreement with one comparable data from the study on Romanian population [18]. On the other hand, the small sample size (32 extracted teeth) was utilized in this study, which can jeopardize generalizability and external validity of the study. Strictly speaking, it can be considered as conflicting evidence. Additionally, Stein [20] found that there was 20.5% positive correlation between age and deviation of apical foramen from the anatomic vertex. So, it should be thought that most of the specimen used in our study may be from young adult.

Approximately, 17% of the specimens was shown as having the detailed anatomy in this study such as lateral canal, accessory canal, and apical delta. This value is deficient when comparing with other studies on Turkish and Japanese populations [16, 17]. The findings from the different populations cannot be adjusted to compare because of unequal distribution of each sample size and different research methods used.

As additional findings, nature of intercanal calcification centers varied from specimen to specimen such as fusiform, islet, and beads. Gani et al. [21] speculated that the calcification center and intercanal communications were associated with the different ages of the individual specimen. So, it seems to be the association or correlation between the covariate, age, and the outcome of interest, in root canal anatomy. We should keep in mind that there is no temporal relationship between these variables. At the same time, we have excluded the covariate and age at the study protocol.

Although Vertucci's classification was widely applied in most of the previous reports of root canal anatomy of the permanent dentition, it has not been agreed as an ideal arrangement in this era. One study reported that 13% of the specimens was not harmonized with Vertucci's classification [22]. Because all categories of root canal anatomy of human dentition found around the world were not included in this classification, additional classes of Gulabivala et al. [9] and new classification protocol are found by Armed and colleagues [23]. As a result, Vertucci's classification has deficiency in content validity. To attain this validity, other categories and calibration of observer were considered in addition to Vertucci's classification in the study protocol to avoid subsequent misclassification bias and observer bias.

There were six classes of internal morphology of permanent mandibular incisors observed in the study. They were Vertucci's type Ι, type ΙΙ, type ΙΙΙ, 1-2-1-2-1, 1-3-2, and 2-3-1 canal configuration. Vertucci's type Ι, ΙΙ, and ΙΙΙ were bound to almost 93% of the sample. And most of the findings may be resulting from the inadequate sample size to fix each and every class. The more the categories, the larger the sample size needed. Small samples indicate imprecision of the results [24], downgrading both internal and external validity of the study. At the opposite end, oversampling can cause unlimited usage of the resources leading to be an ethically inappropriate study. Also, the advantages of in vitro study are internal validity that can be attained by means of aforementioned criteria with controlled experiment, and also, the sample size can be adjusted. Moreover, the potential confounding variables such as age and gender of the subjects can be controlled and even excluded in such cases [25].

As described in protocol, the teeth intended for the study were drawn through extractions or collections of public sector dental clinic, hospitals, and departments in Mandalay Region. So, the results from the study have limited generalizability. They may be poor references of overall Myanmar population. Only random sampling (probability sampling) from the target population can fulfill generalization because every unit of the target population should have an equal opportunity to be involved in the study with a probability. And, there was limited time frame allowed to undertake this study by the Research and Ethic Committee. On the other hand, the more unlimited usage of resource, the weaker the ethical work.

There are several methods to investigate root canal anatomy of permanent dentition other than staining and clearing. Of these, tooth sectioning, CBCT, and the current gold standard, micro-CT, are popular. Tooth sectioning is destructive and ex vivo in spite of being economic. Consequently, CBCT is a useful tool for in vivo and clinical investigation. However, it has low sensitivity to detect detailed anatomy [11]. And, the expense, long processing time, and high learning curve needed may be the drawbacks of micro-CT.

Similarly, the negative consequences of the staining method are unforeseen. First, invasion of ink into the dentinal tubules due to the particle size of ink is small enough to penetrate the tubule [14]. And, decalcification time varied from specimen to specimen due to different mineral contents of teeth from different individuals. So, type ΙΙΙ error may result from interspecimen variation, which may jeopardize study's findings. Fogging of the specimen after clarification is an example of under decalcification. Repeated attempts can waste the resources.

As a result of the study, a summary table was illustrated, which could be also the take-home points for dental clinicians about the evidence of second and third canals in permanent mandibular incisors. And, from the data of this table, second canal anatomy in the sampled Myanmar population comprised 3 in 10 permanent mandibular incisors. This finding coincides with the finding on Caucasian mandibular incisors [7]. More than 1 mandibular incisor out of 10 in Japanese population [17] and 4 of 10 mandibular incisors in a Turkish population [16] disagree the finding of the study.

Because of the three canals and 1-2-1-2-1 configurations being rarely seen, the incidence of the third canal and complex root canal anatomy refreshes as the new knowledge for dental practitioners in Myanmar. Also, 1-2-1-2-1 canal configuration was detected as the third mode in this study. Although most of Myanmar mandibular incisors been having one canal and one foramen, the remaining multifaceted anatomy can become the culprit of endodontic failure. Particularly, up to 70% of apical foramina of mandibular incisors was drained at the center of the root. This may take illusion in working length determination in endodontic practice because of most studies [7, 16, 17] pointing out lateral allocation in such case. However, rarely seen intricate anatomy in this study can benefit for dental practitioners. As an exploratory analysis, bead type of intercanal calcifications out of three, such as fusiform, islet, and bead, can uphill the coronal access of endodontic treatment to become difficult. All of the above statement is concerned with clinical implication.

The future replicated study can obey generalizability by means of sample size calculation and should be allowed to take the adequate time frame. Moreover, a cross-sectional analytical design on evidence of the second canal between permanent mandibular central and lateral incisors in a Myanmar population can be considered as future research scenario. Finally, it should be taken into consideration that in vivo and state-of-the-art research with more statistical power and sample size than this study may become a demand. So, the above statement is implication of research.

3 out of 10 sampled Myanmar mandibular incisors in Mandalay Region had second canal anatomy. So, the proposed null hypothesis, that there was no second canal anatomy in Myanmar permanent mandibular incisors in Mandalay Region, was rejected.

## 5. Conclusion

Vertucci's type I, the most common type of root canal anatomy of a sampled Myanmar mandibular incisor in Mandalay Region, was followed by type III. In contrast to the previous findings, there was the slight burden on unusual root canal anatomy such as 1–3-2, 2-3–1, and 1-2-1-2-1. The findings reflected that types of Vertucci's classification, other than type Ι and type ΙΙΙ, were poorly investigated in this study. The controversial evidence was central allocation of apical foramen opening of Myanmar permanent mandibular incisors commonly found. Then, lateral canal, accessory canal, and apical delta of the population were less abundant than the findings on other populations. For the clinician, the evidence of the second canal or third canal in Myanmar population comprised 3 in 10 permanent mandibular incisors. As an additional finding, there were three types of intercanal calcifications such as fusiform, islet, and bead in this study. However, the reader should interpret that small sample size reflects the findings of this study, and enough time interval to allow adequate sample size is needed.

## Figures and Tables

**Table 1:** Vertucci's classification [7].

Type I	One canal from pulp cavity to the apex (1-1 configuration)
Type II	Two dividing canals converging short of the apex as one canal (2-1 configuration)
Type III	A single canal diverging into two and then reunite as one (1-2-1 configuration)
Type IV	Two canals throughout the root (2 configuration)
Type V	A single orifice and drains as two canals (1-2 configuration)
Type VI	Two canals binding in the middle of the root and separate as two (2-1-2 configuration)
Type VII	One canal which separate, converge, and open as two separate canals to the apex (1-2-1-2 configuration)
Type VIII	Three canals throughout to the apex (3 configuration)

**Table 2:** Baseline distribution of types of mandibular incisors.

Types of teeth	Number of teeth (*n*)	Percentage (%)
Permanent mandibular central incisors	58	60.4
Permanent mandibular lateral incisors	38	39.6

**Table 3:** Root canal anatomy of mandibular incisors according to Vertucci's classification.

Vertucci's classification	Type Ι	Type ΙΙ	Type ΙΙΙ	Type ΙV	Type V	Type VΙ	Type VΙΙ	Type VΙΙΙ	1-3-2	2-3-1	1-2-1-2-1
Permanent mandibular central incisors	42	1	10	—	—	—	—	—	1	—	4
Permanent mandibular lateral incisors	26	—	11	—	—	—	—	—	—	1	—
Total (percentage)	68 (70.8%)	1 (1.04%)	21 (21.88%)						1 (1.04%)	1 (1.04%)	4 (4.17%)

**Table 4:** Locations of apical foramen in mandibular central and lateral incisors.

Types of teeth	Locations of apical foramen	
	Central location, *n* (%)	Lateral location, *n* (%)
Permanent mandibular central incisors	40 (41.66%)	18 (18.75%)
Permanent mandibular lateral incisors	27 (28.12%)	11 (11.46%)
Total	67 (69.78%)	29 (30.21%)

**Table 5:** Distribution of lateral canal, accessory canal, and apical delta in mandibular central and lateral incisors.

Types of teeth	Lateral canal, *n* (%)		Accessory canal, *n* (%)		Apical delta, *n* (%)	
	Presence	Absence	Presence	Absence	Presence	Absence
Permanent mandibular central incisors	2 (2%)	56 (58.33%)	5 (5.21%)	53 (55.21%)	3 (3.08%)	55 (57.29%)
Permanent mandibular lateral incisor	4 (4.13%)	34 (35.41%)	2 (2.08%)	36 (37.51%)	1 (1.00%)	37 (38.54%)
Total	6 (6.25%)	90 (89.75%)	7 (7.29%)	89 (92.71%)	4 (4.17%)	92 (95.83%)

**Table 6:** Distribution of number (%) of root canal in permanent mandibular central and lateral incisors (summary table).

Types of teeth	Numbers of canals		
	One canal *n* (%)	Two canals *n* (%)	Three canals *n* (%)
Permanent mandibular central incisors	42 (43.75%)	15 (15.63%)	1 (1.04%)
Permanent mandibular lateral incisors	26 (27.09%)	11 (11.46%)	1 (1.04%)
Total	68 (70.84%)	26 (27.09%)	2 (2.08%)

## Data Availability

The data used to support this study are available at 10.17632/6w7gdtb3zx.

## References

[1] Ingle J. I., Bakland L. K. (2002). *Endodontics*.

[2] Allen R. K., Newton C. W., Brown C. E. (1989). A statistical analysis of surgical and nonsurgical endodontic retreatment cases. *Journal of Endodontics*.

[3] Han T., Ma Y., Yang L., Chen X., Zhang X., Wang Y. (2014). A study of the root canal morphology of mandibular anterior teeth using cone-beam computed tomography in a Chinese subpopulation. *Journal of Endodontics*.

[4] Valenti-Obino F., Nardo D. D., Quero L. (2019). Symmetry of root and root canal morphology of mandibular incisors: a cone-beam computed tomography study in vivo. *Journal of Clinical and Experimental Dentistry*.

[5] Altunsoy M., Ok E., Nur B. G., Aglarci O. S., Gungor E., Colak M. (2014). A cone-beam computed tomography study of the root canal morphology of anterior teeth in a Turkish population. *European Journal of Dentistry*.

[6] Magdalena L. N. B., Nogueira B. C. L., Carolina N. F. (2017). Root and canal morphology of permanent mandibular incisors. *International Journal of Odontology & Stomatology*.

[7] Vertucci F. J. (1984). Root canal anatomy of the human permanent teeth. *Oral Surgery, Oral Medicine, Oral Pathology*.

[8] Versiani M. A., Pereira M. R., Pécora J. D. (2018). Chapter 7: root canal anatomy of maxillary and mandibular teeth. *The Root Canal Anatomy in Permanent Dentition*.

[9] Gulabivala K., Aung T. H., Alavi A., Ng Y.-L. (2001). Root and canal morphology of Burmese mandibular molars. *International Endodontic Journal*.

[10] Moe M. M. K., Jung-Hong H., Myoung-Uk J. (2017). Anatomical profile of the mesial root of the Burmese mandibular first molar with Vertucci’s type IV canal configuration. *Journal of Oral Science*.

[11] Sousa T. O., Hassan B., Mirmohammadi H. (2017). Feasibility of cone-beam computed tomography in detecting lateral canals before and after root canal treatment: an ex vivo study. *Journal of Endodontics*.

[12] Iqbal A. (2016). The factors responsible for endodontic treatment failure in permanent dentition of the patients reported to the College of Dentistry, the University of Aljouf, Kingdom of Saudi Arabia. *Journal of Clinical and Diagnostic Research*.

[13] Bloom B. R., Michaud C. M., La Montagne J. R. (2006). Chapter 4: priorities for global research and development of interventions. *Disease Control Priorities in Developing Countries*.

[14] Barrington C., Balandrano F. (2018). Chapter 5: diaphanization techniques in the study of root canal anatomy. *The Root Canal Anatomy in Permanent Dentition*.

[15] Orwin R. G., Cooper H., Hedges L. V. (1994). Evaluating coding decisions. *The Handbook of Research Synthesis*.

[16] Kartal N., Yanıkoğlu F. Ç. (1992). Root canal morphology of mandibular incisors. *Journal of Endodontics*.

[17] Miyashita M., Kasahara E., Yasuda E. (1997). Root canal system of the mandibular incisor. *Journal of Endodontics*.

[18] Scarlatescu S., Didilescu A. C., Stratul S. (2010). Root canal morphology of mandibular central incisors in south-eastern Romanian population: endodontic and periodontal implication. *TMJ*.

[19] Martins J. N. R., Marques D., Mata A., Caramês J. (2017). Root and root canal morphology of the permanent dentition in a Caucasian population: a cone-beam computed tomography study. *International Endodontic Journal*.

[20] Stein T. J., Corcoran J. F. (1990). Anatomy of the root apex and its histologic changes with age. *Oral Surgery, Oral Medicine, Oral Pathology*.

[21] Gani O. A., Boiero C. F., Correa C. (2014). Morphological changes related to age in mesial root canals of permanent mandibular first molars. *Acta Odontol Latinoam*.

[22] Filpo-Perez C., Bramante C. M., Villas-Boas M. H., Húngaro Duarte M. A., Versiani M. A., Ordinola-Zapata R. (2015). Micro-computed tomographic analysis of the root canal morphology of the distal root of mandibular first molar. *Journal of Endodontics*.

[23] Ahmed H. M. A., Versiani M. A., De-Deus G., Dummer P. M. H. (2017). A new system for classifying root and root canal morphology. *International Endodontic Journal*.

[24] Kelley K., Maxwell S. E., Rausch J. R. (2003). Obtaining power or obtaining precision. *Evaluation & the Health Professions*.

[25] Faggion C. M. (2012). Guidelines for reporting pre-clinical in vitro studies on dental materials. *J Evid Base Dent Pract*.

